# Healthcare Digitalization and Pay-For-Performance Incentives in Smart Hospital Project Financing

**DOI:** 10.3390/ijerph17072318

**Published:** 2020-03-30

**Authors:** Roberto Moro Visconti, Donato Morea

**Affiliations:** 1 Department of Business Management, Catholic University of Sacred Heart, Via Ludovico Necchi, 7, 20123 Milan, Italy; 2 Faculty of Economics, Universitas Mercatorum, Piazza Mattei, 10, 00186 Rome, Italy

**Keywords:** public–private partnerships, internet of medical things, digital innovation, healthcare sustainable development, patient-centered care, mHealth, healthcare bottlenecks, coronavirus, results-based-financing

## Abstract

This study aims to explore the impact of healthcare digitalization on smart hospital project financing (PF) fostered by pay-for-performance (P4P) incentives. Digital platforms are a technology-enabled business model that facilitates exchanges between interacting agents. They represent a bridging link among disconnected nodes, improving the scalable value of networks. Application to healthcare public–private partnerships (PPPs) is significant due to the consistency of digital platforms with health issues and the complexity of the stakeholder’s interaction. In infrastructural PPPs, public and private players cooperate, usually following PF patterns. This relationship is complemented by digitized supply chains and is increasingly patient-centric. This paper reviews the literature, analyzes some supply chain bottlenecks, addresses solutions concerning the networking effects of platforms to improve PPP interactions, and investigates the cost–benefit analysis of digital health with an empirical case. Whereas diagnostic or infrastructural technology is an expensive investment with long-term payback, leapfrogging digital applications reduce contingent costs. “Digital” savings can be shared by key stakeholders with P4P schemes, incentivizing value co-creation patterns. Efficient sharing may apply network theory to a comprehensive PPP ecosystem where stakeholding nodes are digitally connected. This innovative approach improves stakeholder relationships, which are re-engineered around digital platforms that enhance patient-centered satisfaction and sustainability. Digital technologies are useful even for infectious disease surveillance, like that of the coronavirus pandemic, for supporting massive healthcare intervention, decongesting hospitals, and providing timely big data.

## 1. Introduction

This study aims to explore the impact of healthcare digitalization on smart hospital project financing (PF) fostered by Pay-for-Performance (P4P) incentives. The background is represented by a consolidated trend of aging patients and population growth, which contributes importantly to the rise in healthcare costs. The main drivers for growth in healthcare costs, besides an aging population, are represented by health insurance, and—mainly—technological progress [1].

As Deloitte indicates, financial sustainability, care delivery, patient centricity, digital transformation, and regulatory compliance are at the top of the agenda [2]. Additionally, global healthcare expenditure is projected to increase at an annual rate of 5.4 percent between 2017–2022, from USD $7.724 trillion to USD $10.059 trillion [2]. Despite common perceptions, the bulk of health expenditure growth is not due to population aging per se, but to the increase in demand for new medical technologies that improve and/or extend life as real per-capita incomes grow [3,4].

Within this evolving framework, technology is a double-edged sword, since it increases healthcare expenditure [5] but can also bring savings and quality-of-life improvements [6]. The relationship between medical technology and spending is complex and often conflicting. The impact of technology on costs differs across technologies, in that some (e.g., cancer drugs, invasive medical devices) have significant financial implications, while others are cost-neutral or cost-saving [7]. Medicine in the 21^st^ century is increasingly dependent on technology. Unlike in many other areas, the cost of medical technology is not declining, and its increasing use contributes to spiraling healthcare costs. Many medical professionals equate progress in medicine to the growing use of sophisticated technology, which is often expensive and beyond the reach of the average citizen [8].

Whereas technology concerning tangible items, such as diagnostic equipment or physical infrastructure, has an uncertain cost–benefit trade-off, digital investments have a shorter payback, are typically cheaper, and show sounder benefits relative to their costs. Digital healthcare is a major driver of innovation, growth, and competitiveness. Digital technology is reported to reduce healthcare costs by 7 to 11 percent [9,10]. Cashless (digital) payment savings account for 0.5% of gross domestic product (GDP) [11]. To the extent that GDP can roughly be assimilated to revenue, and considering an incidence of some 80% of operating costs (EBIT/revenues), the estimated operating cost reduction due to cashless payments is around 0.4%. An international comparison of health prices across countries can improve the private competition for international public–private partnership (PPP) tenders [12].

Further savings may be envisaged due to the synergistic interaction of intangibles (e.g., big data and the Internet of Medical Things (IoMT). These bundled resources feed interoperable cloud databases linked to digital platforms [13] with information secured by blockchains and interpreted with artificial intelligence algorithms [14]). Digital investments actively interact with physical technologies (e.g., for the eHealth transmission of scans or lab exams) and contribute to increasing their added value.

Digital investments in infrastructural healthcare projects are often undertaken with PPPs, where the public player sets the goals and necessities, whereas the private actor (backed by its sponsoring banks) provides the technological expertise and takes on most of the investment’s risk. Eurostat’s definition of a PPP requires that a government entity is the direct source of most of the revenues that the partner is entitled to receive under the contract. This is the case if the demand for or use of the asset originates from the government entity itself (e.g., a hospital paid for by a government entity on an availability basis) [15].

Smart hospitals represent the latest frontier of healthcare impact investments. Technological features are so advanced that public authorities hardly possess the know-how to conceive, build, and operate them [16]. Many public bureaucrats are also technophobic. The increasing pace of change in healthcare technologies and policies has generated increased interest in the future adaptability of the physical infrastructure that supports health services [17]. Technological investments are often made possible by public–private cooperation within healthcare PPP agreements backed by project finance (PF) patterns. The introduction of health information technologies (HIT) increases organizational performance, including productivity enhancement and cost reductions. HIT can contribute to hospital profitability, for instance, by reducing paper chart pulling and document transportation, reducing medical errors, and potentially lowering medical liability costs as well as decreasing back-office expenses [18,19]. These technological advances hence contribute to making the model sustainable and profitable for all the stakeholders.

Consistent with this evolving framework, this paper will concentrate on some key aspects that concern the digital impact on infrastructural healthcare investments that face increasing public budget pressures. As it will be shown in the methodological section, the study focuses on the estimation of the potential digital savings accruing to the private player. These savings must then be shared with the public player (to avoid undeserved private rents) and returned, as far as possible, to the patients, in the form of lower fees and improved care. Part of the saving should also be reinvested in expensive “hard” (nondigital) technology. This should ease capital rationing concerns that represent a significant bottleneck in healthcare investments, fostering long-term socio-economic sustainability. This sequential model will be tested with an empirical case.

This paper is, to the authors’ best knowledge, innovative, and it might shed some light both on traditional stakeholder relationships, re-engineered around digital platforms, and on the specific healthcare sector, looking for patient-centered satisfaction and sustainability.

The study is organized as follows. An interdisciplinary literature review, showing the research state of the art and gaps, is contained in Section 2. Section 3 describes the methodology, combining the study’s motivation and purpose with its background. The research question concerns an estimate of the impact of the digital savings to soften supply chain bottlenecks. A corollary aspect is represented by the (optimal) public–private–patient sharing of savings with P4P schemes. The methodology also contains an outline of the investigation area and a description of the consequential reasoning that it brings to the empirical case. Section 4 analyzes some supply chain bottlenecks that are part of the study background, and whose softening is an ancillary component of the motivation and purpose of the paper. Section 5 addresses specific solutions to some healthcare bottlenecks, through an analysis of value co-creation patterns based on digital health platforms. Digitalization and networking of key stakeholders will be analyzed as an ideal interactive background for these solutions. Section 6 analyzes the cost–benefit analysis of digital health with an empirical case. The economic and financial impact of digitalization will be tested with a sensitivity analysis on a sample of healthcare PPP investments. The discussion (Section 7) and the conclusions (Section 8) critically examine and summarize the main findings. Some policy implications (P4P sharing of digital savings; impact of digitalization on PPP contracts) will be analyzed, together with practical recommendations and tips for further research.

## 2. Literature Review

This study considers applications to the specific healthcare industry. Since the topic is highly interdisciplinary, this synthetic literature review will consider the main streams that deal with each field, to find out how they may interact, and what are some possible research gaps. The four main subdivisions are: healthcare PPP/PF investments; pay-for-performance incentives; digital platforms nurturing eHealth/mobile health (mHealth) applications; patient-centered issues.

As a result, PPP healthcare (interacting with networked digital platforms and stimulated by patient-centered governance concerns) can become “smart”, being driven by technological sustainability.

The literature streams are very different and, in many aspects, innovative. It is hard to find a common denominator that jointly examines the application of digital platforms on traditional PPP healthcare to make investments smart, using P4P incentive schemes.

The original idea behind this study, consistent with its background and motivation, is that technological (smart) PPP healthcare investments may be consistently improved. To do so, traditional PPP models should be scaled up with networked digital platforms. P4P/RBF schemes may then be used to share productivity gains, eventually benefitting patients. The literature synthetized here shows the interdisciplinary boundaries of the study.

### 2.1. Healthcare PPP/PF Investments

PPP research is heterogeneous, covers different interdisciplinary topics and is disseminated in many journals [20]. PPPs have become popular tools to deliver infrastructure and public services around the world [21,22,23,24,25]. A bibliometric analysis of PPP and PF literature is contained in [26].

Traditionally, public–private partnerships (PPPs) have been most often exploited to foster hard infrastructural investments such as transportation, energy, water, and solid waste. However, during the last decades, there has been a sharp rise—predominantly within Europe—in PPPs to deliver social infrastructure, mainly healthcare infrastructure. European PPP Expertise Centre (EPEC) [27,28] show PPP healthcare statistics for Europe.

PPPs have become popular worldwide as a way of improving healthcare service delivery, [29,30], and are typically associated with PF [31]. Healthcare PPPs have the potential to generate several benefits, including (i) better investment decisions, (ii) more efficient infrastructure delivery, and (iii) higher quality health services. However, PPPs are also associated with additional transaction and financing costs and may give rise to affordability challenges [32].

While some forms of PPPs are a feature of hospital construction and operation in all countries with mixed economies, there is increasing interest in a model in which public authority contracts with a private company to design, build and operate an entire hospital [33].

Governments around the world, but especially in Europe, have increasingly used private sector involvement in developing, financing, and providing public health infrastructure and service delivery through PPPs. Reasons for this uptake are manifold, including rising expenditures for refurbishing, maintaining and operating public assets, and increasing constraints on government budgets stifle, prompting innovation through private-sector acumen and aiming for better risk management [34].

There is a recognized need to incorporate sustainability considerations in infrastructure projects delivered through PPPs. Public authorities are encouraged to find innovative solutions to foster sustainable welfare and increasingly look for private partners through PPP schemes [35].

### 2.2. Pay-For-Performance Incentives

Performance-based financing (or P4P) is a financing mechanism that gives healthcare providers (facilities or health workers) financial payments based on the achievement of predetermined targets, goals, or outputs after being verified for quality [36].

Payment is made conditional on measurable actions to foster results-oriented management. P4P is an umbrella term that includes pay-for-performance contracts with healthcare providers, output-based aid, and conditional cash transfers and other demands. 

The P4P model should adequately consider a system of economic incentives for private players acting together with public actors in a PPP/PF agreement. This coordination is consistent with a PPP model that is widely used in healthcare [37]. P4P is complementary to mHealth and remunerates digital efforts and convergence to electronic health records. The creation of an information feedback mechanism can move healthcare delivery towards results-based practice and help make more efficient use of scarce resources [38].

Experimental P4P-based payment systems have led to rapid qualitative and quantitative improvements in access to healthcare [39]. P4P is a valuable tool for donors to guarantee transparency and accountability throughout the healthcare supply chain, which subsidizes local healthcare providers for achieving specific benchmarks. 

P4P has become a popular approach to increase efficiency in healthcare. Evidence about the effects of P4P in healthcare remains, however, mixed [40,41,42].

Effective P4P program measures should be aligned with organizational goals, and incentive structures should be carefully considered. Factors such as a strong infrastructure and public reporting may have a large influence [43]. Programs are, however, very heterogeneous in their design, and they do not account for a large part of the hospital budget [44].

Although traditional fee-for-service (FfS) reimbursement is still a large percentage of income for hospitals, the shift towards payment for value-based healthcare programs is accelerating rapidly. In P4P programs, hospitals are required to pay attention to a broad array of factors which they are not incentivized to address in traditional FfS systems. There are two basic types of P4P designs being deployed for hospitals. In the first, payers reduce global FfS payments and use the funds to reward hospitals based on how well they perform across process, quality, and efficiency measures. In the second, hospitals are penalized financially for sub-par performance, and the penalties are either translated into direct cost savings for payers or are used to generate an incentive pool [45].

### 2.3. Digital Platforms Nurturing eHealth/mHealth Applications

Literature reviews on digital platforms are contained in Baldwin and Woodard (2009), Parker et al. (2017), Basole and Carla (2011), Srinivasan and Venkatraman (2017), Asadullah et al. (2018), Constantinides et al. (2018), Cremona et al. (2014), and in Sutherland and Jarrahi (2018), who analyze sharing economy platforms [46,47,48,49,50,51,52,53].

Spagnoletti et al. [54] define a digital platform as “a building block that provides an essential function to a technological system and serves as a foundation upon which complementary products, technologies, or services can be developed”.

eHealth devices are a specific segment of digital platforms, operating in a sensitive industry [55,56]. Innovative digital health devices allow easy and accurate characterization of health and disease [57,58]. Technological advancements and the miniaturization of diagnostic instruments to modern smartphone-connected and mHealth devices such as the iECG, handheld ultrasound, and lab-on-a-chip technologies have led to increasing enthusiasm for patient care with promises to decrease healthcare costs and to improve outcomes [56].

Technology is used extensively to provide and deliver healthcare worldwide. eHealth (the application of information, computer or communication technology to some aspects of health or health care) is viewed as essential for solving the problems facing healthcare systems (Expert Panel on Effective Ways of Investing in Health, 2019) of increasing demand, due to an aging population and improved treatments, and limited resources [59,60]. However, although there is widespread agreement about the importance and potential benefits of eHealth, the realization of these benefits has often been slower than anticipated, usually because of difficulties with implementation [61].

Digital therapeutics are technology-based solutions that have a clinical impact on disease comparable to that of a drug. They primarily use consumer-grade technology such as mobile devices, wearable sensors, big data analytics, and behavioral science. They can be delivered through web browsers, apps, or in conjunction with medical devices [62,63]. They can also be deployed in real time and at scale, which is critical for intervention in chronic diseases [64].

### 2.4. Patient-centered Issues

The consideration that hospitals should be patient-centric seems obvious but is, in practice, often underestimated. Accordingly, value-based health care is a framework for restructuring health care systems around the globe with the overarching goal of value for patients [65]. Patient experience is one of the key domains of value-based purchasing that can serve as a measure of quality and be used to improve the delivery of health services [66]. Patient- and family-centered care interventions are increasingly being implemented in various settings for enhancing the quality of healthcare [67] and its indicators [68].

Quality of care is improving in terms of safety and effectiveness, but more attention should be placed on patient-reported findings and experiences. A deeper understanding of the quality of care requires measuring what matters to people. However, few health systems routinely ask patients about the outcomes and experiences of their care. Preliminary results show improvements in patient-reported outcomes. For example, following a hip replacement, an individual’s quality of life—in terms of mobility, self-care, activity, pain, and depression—improved on average by around 20% [69].

Patient empowerment positively influences value co-creation, which, in turn, is positively related to patient satisfaction [70]. A functional life for the patient is the main goal of patient-centeredness, which involves empathy, respect, engagement, communication, shared decision-making, holistic focus, individualized focus, and coordinated care [71].

Smart hospitals are likely to have a significant impact on customer satisfaction and patient-centered governance issues [6]. The networked digital platforms analyzed in this study can be a catalyzer of much-wanted PPP efficiency gains.

## 3. Methodology

Within the framework described in the introduction, the research question of this study, consistent with its background and purpose, will try to:

1. Estimate the potential impact of digital savings on the economic and financial margins of a private special purpose vehicle (SPV);
2. Show how these digital extra-gains can be shared among the key stakeholders with pay-for-performance (P4P) or results-based financing (RBF) contractual schemes.

The model has substantial socio-economic sustainability consequences, as anticipated in the introduction.

A sort of syllogism, coherent with the research question, can be synthesized as follows:

1. Demand for healthcare technologies is growing but expensive, facing public budget constraints;
2. “Digital” technology is, however, cheaper and quicker to cash in;
3. Therefore, digital technology is easier to adopt, and P4P/RBF schemes incentivize private—public value co-creation (which can partially be used to fund otherwise unaffordable “hard” technologies).

This consequential reasoning will be applied to infrastructural PPP investments in healthcare that are traditionally capital- and labor-intensive. Savings ease public affordability, private bankability, and the overall sustainability of healthcare ecosystems.

This thesis is consistent with the well-known health-led growth hypothesis that claims a positive correlation between health expenditure and economic growth [72]. If healthcare expenditure becomes affordable, it can ignite a win–win spiral where part of the economic growth is dedicated to financing further healthcare investments that will catalyze additional growth.

The research question illustrated above be given a tentative answer, considering an empirical case where a template healthcare PPP investment is subject to a sensitivity analysis, to show the potential (positive) impact of digitalization on the economic and financial margins of the SPV. These economic and financial savings must then be shared between the private and the public part (including the patients), following contractual agreements that include P4P provisions. Whereas P4P has been extensively used in healthcare in the last twenty years (see Section 2.2. for bibliographical references), little if any attention has been paid to technological investments and the partitioning of their savings. 

The premises of the investigated issues are given by a theoretical framework where traditional healthcare bottlenecks (examined in Section 4) can be partially softened with digitalization. A cost–benefit analysis of digital health investments (analyzed in Section 6) will show how digital platforms can promote—through their networking properties—economic and financial savings due to their scalability. Section 6 illustrates an empirical case of an economic and financial plan (with data taken from a sample or real PPP infrastructural healthcare investments) where digital savings are considered. A sensitivity analysis, incorporating incremental savings in operating costs, will show different patterns of sustainability. It will be demonstrated that digital savings bring higher economic and financial margins that improve traditional PF parameters (net present value, internal rate of return, payback period, etc.).

Subdivision of the savings can then be carried on following the P4P/RBF patterns. Whereas cost–benefit analysis is traditionally used to assess healthcare investments [73], the implications of digitalization within a PPP infrastructural framework have hardly been investigated. This topic therefore seems innovative, and this also emerges from the literature review.

## 4. Healthcare Supply Chain Bottlenecks

An analysis of the primary healthcare supply chain bottlenecks represents the propaedeutic background to the examination of the impact of digital technology, consistent with the research question and the study purpose illustrated in Section 1 and Section 3 [74]. The target is to show that digital investments can bring savings that may conveniently be shared by the private and public players in P4P contractual agreements.

The healthcare supply chain has attracted attention by scholars, researchers, government officials, and providers as one of the main tools in their effort to manage healthcare costs and improve quality at the same time [75,76]. A traditional supply chain is a network between a company and its suppliers to produce and distribute a specific product to the final buyer. Healthcare is, however, not simply based on supply and demand. Affordability, sustainability, and quality of patient care complement the economic features of standard supply chains. Organizational performance is affected by healthcare supply chain management [77].

Healthcare providers are required to deliver high-quality medical services to their customers. Since most of their budgets are spent on high-cost medical equipment and medicines, there is a pressing need for them to optimize their supply chain activities such that high-quality services can be provided at lower costs. Relatedly, medical equipment and devices generate massive amounts of unused data. Big data analytics is proven to be helpful in forecasting and decision-making, and, hence, can be a powerful tool to improve healthcare supply chains [78]. Additionally, big data represent a core input factor for digitalization processes.

Global economic growth is creating new demand for affordable and effective healthcare products, especially in emerging economies. Better supply chain performance will provide significant strategic benefits [79]:

1. It can reduce costs, shortening manufacturing lead times, slashing inventory levels across the value chain, and cutting product obsolescence;
2. It can improve access, reducing drug and device shortages;
3. It can reinforce safety, making it harder to counterfeit products and reducing the human and financial tolls of medication errors. Blockchain technology can strengthen this popular strategy [80];
4. It can favor the change of status of patients, transforming them (whenever possible, e.g., in the absence of acute contingencies) from inpatients to outpatients and, eventually, home patients [81].

The deficient performance of healthcare providers plagues the delivery of health services, especially in low- and middle-income countries [82,83]. Additionally, the overall cost of healthcare is increasing, with an aging population and higher pressure for quality of care. A synthetic analysis of healthcare supply chain bottlenecks is preliminary to some technological proposals, within a PPP framework where smart healthcare infrastructural projects are considered. Best practices may help to overcome the most common bottlenecks in the chain [84].

The biggest failure in healthcare is the tendency to overlook the fact that the consumer is the patient. Consequently, all stakeholders (e.g., governments, private payers, MedTech and pharma companies, and providers) almost always ignore consumer-oriented value propositions. Instead, they focus on creating and delivering their value propositions with one another. This has resulted in healthcare systems around the world failing to deliver on the triple aim of affordable, accessible, and effective healthcare—with the most pronounced failures occurring in the United States [85].

According to [86], when effective, the supply chain is the “backbone” for access to safe and effective health products and supports the goals of eliminating AIDS, tuberculosis, and malaria, ending childhood vaccine-preventable deaths, and ensuring universal access to reproductive health services. An efficient supply chain also safeguards the significant financial investments in the procurement of health products by donors and governments. Yet, public health supply chains are often suboptimal and unable to support the achievement of the broader health goals of a country, due to a combination of failures relating to people, processes, technology, or resources.

Supply-chain-critical issues have been specifically examined in the healthcare sector [75,87,88,89,90] and may, for instance, concern:

- Last-mile unavailability or difficulties in delivering health services;
- First-mile (health center) data and human resources (HR) shortages;
- Paper/non-digital data;
- Data-driven performance management;
- Governance and accountability drawbacks;
- Sustainable human capacity/local capacity building;
- Resource mobilization and supply chain operations financing;
- Lack of integrated diagnostic services;
- Public budgetary constraints.

The challenge is to make technological and process improvements in all the areas mentioned above, while at the same time balancing the cost trade-offs. The exchange of small and big data (isolated or, respectively, gathered information), goods, and services along networked digital chains may substantially contribute to softening healthcare bottlenecks, as it will be shown in the next sections.

Another issue is represented by MedTech potentialities and costs, with a challenging trade-off. The medical technology industry is undergoing a period of fundamental change. On the one hand are changing demographics in most parts of the world and the increasing prevalence of chronic diseases driving the demand for high-quality medical devices, diagnostic and imaging equipment, and innovative eHealth solutions. On the other hand, healthcare expenditure is increasingly curbed by strained public budgets and austerity measures [91].

Several bottlenecks slow down the transition from conventional to personalized medicine. They may be represented by generation of cost-effective high-throughput data, hybrid education and multidisciplinary teams, data storage and processing, data integration and interpretation, and individual and global economic relevance [92].

mHealth is a sub-segment of eHealth that refers to the use of mobile communication devices for health services, information, and data collection. mHealth softens some of the healthcare supply chain bottlenecks described above, such as infrastructural deficiencies, limited access to medical care, and the shortage of skilled healthcare workers.

The uneasy application of digital health in Italy shows which are the signs of progress made but also the catch-up potential. According to Osservatorio Innovazione Digitale in Sanità [93], the expenses for digital healthcare in Italy have grown in 2018 by 7% (2% in 2017) with investments concentrated in the electronic medical record and the department systems. Eighty-five percent of general medical doctors (81% of specialized doctors) use email to communicate with patients (64% of general doctors and 57% of specialized doctors use WhatsApp). Online booking is used by 11% of patients and 7% pay online. A total of 41% of citizens use wearables, smartwatches, or other devices to monitor their health. Image diagnostics is digitalized in 88% of cases (86% of lab analyses). Digital apps are widely used in radiology (84%), but less in ultrasound (40%), ECG/EEG (33%), or digital pathology apps (7%). The use of mobile applications (m-apps), blogs, or online health searches for information is continuously growing. eProcurement of digital solutions is still a bottleneck due to insufficient awareness of its potential and to legal obstacles. More comprehensive application of digital health in Italy may substantially improve the quality of life of some 24 million people (40% of the population) that suffer from chronic diseases. Italy currently invests in digital healthcare only €22 per citizen (compared to €70 per citizen in Denmark; €60 per citizen in the UK and €40 per citizen in France). Digitalization eases the exchange of information, sharing data in real-time, and permitting instant booking or access to digital health records [94,95]. Artificial intelligence is also starting [96].

## 5. Networking effects, scalability of digital platforms and Healthcare PPP interactions

Digital platforms intrinsically incorporate scalability properties that can be levered if the platform is positioned within a PPP network. Platforms catalyze the interaction of private and public stakeholders, fostering value co-creation patterns. Technology interacts with digital platforms and supply/value chains [97], to get to smart healthcare PPPs.

These considerations are consistent with the background and purpose of the paper, as shown in Section 1 and Section 3. The examination of the impact of digital platforms also corroborates the research question and shows, from a theoretical perspective, what the premises of digitalization gains are.

**Figure 1:**
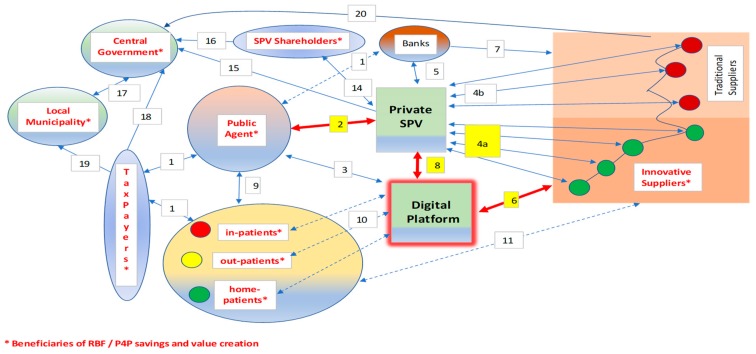
Digital Public-Private Partnership (PPP) management with Pay-for-Performance (P4P)/Results-Based Financing (RBF).

The socio-economic impact of digital platform implementation can foster the overall sustainability of the healthcare ecosystem. Digital actions or strategies that improve patient-centricity may include:

a) Digital scalability [98,99];
b) Electronic health records;
c) MedTech applications;
d) Business-to-business (B2B) auctions conducted through digital platforms, improving the interaction between the SPV and its innovative suppliers (as shown in Figure 1);
e) Healthcare analytics;
f) M-apps for medical access and patient feedback;
g) Disease management and 24/7 surveillance;
h) Personalized/precision medicine;
i) Telemedicine, eHealth and mHealth.

Table 1 (elaborated by the authors, and inspired by several sources [81,83,84,86]) illustrates some action or strategy to co-create value, consistent with a patient-centric approach. A survey of value co-creation is contained in [100].

A complementary strategy may start from actions that are not necessarily related to bottlenecks or inefficiencies but rely on innovative technological approaches that rotate around digital platforms and their applications. The main factors that interact with digital platforms are possibly represented by input parameters proxied by the Internet of Things (IoT) and big data (e.g., transmissible health parameters through wearable devices), and by processing algorithms that use artificial intelligence and deep/machine learning patterns. This process may be conveniently adjuvated by blockchain validation, cloud storage, interoperable databases or other devices and smart technologies, whenever applicable.

The networked links among the main stakeholders are synthesized in Figure 1.

The links among the stakeholding nodes concern the management phase (since, during the construction, P4P/RBF schemes are uneasy about conceiving) and follow these patterns.

1. The public agent makes contractual payments to the bank on behalf of the SPV at stated milestones of the public-to-private concession (remuneration for “cold” services rendered by the private SPV to the public agent; availability payments, consisting of a fee structure in which the public agency makes payments under the relevant agreement to the private-sector party once the project or facility is made available for use);
2. The compensation of the SPV partially depends on P4P/RBF;
3. The digital platform connects the nodes 24/7 (not only the public agent) acting as a replica node for multilayer interactions;
4. The SPV buys products and services from its suppliers: innovative providers (green nodes—4a links) may be additionally rewarded for participation with RBF proceeds;
5. The SPV receives residual payments from the bank (remuneration after bank debt service);
6. The suppliers participate in eAuctions [101] mastered by the SPV (step 4) through the digital platform;
7. The bank pays suppliers on behalf of the SPV;
8. The SPV interacts 24/7 with the digital platform to coordinate eAuctions and exchange information; RBF is enhanced and monitored digitally;
9. The public agent that runs the hospital is continuously coordinated with the "clients" following a patient-centric approach that aims to maximize value for money and cures;
10. Patients interact (in different ways) with the digital platform (e.g., through wearables, online bookings, etc.);
11. Patients may interact with suppliers (e.g., exchanging feedback);
12. Patients represent a sub-set of the general taxpayers and pay with a ticket part of the healthcare costs;
13. The public agent receives residual funds from taxation if direct revenues are insufficient to cover costs fully;
14. The shareholders that control the SPV interact with it to provide capital and subordinated debt and to receive dividends;
15. The SPV pays taxes (mainly) to the central government, based on its positive tax base during the management phase;
16. The SPV shareholders (usually represented by one or more holding/construction/management company) pay taxes on dividends and other incomes;
17. Part of the tax collected by the central government is attributed to local municipalities (regions, provinces, etc.) to finance local healthcare;
18. The central government collects state taxes from taxpayers;
19. Taxpayers pay local tributes, contributing to the budget of municipalities;
20. The suppliers of the SPV pay taxes (according to a tax base calculated on their positive economic margins), mainly to the central government.

The interpretation of PPP interactions following the network theory [102,103] is—to the authors’ knowledge—innovative. Section 6.1. will contain some sensitivity analyses of digital health benefits.

## 6. The Impact of Digitalization on Healthcare PPP Sustainability

The research question, illustrated in Section 3, concerns the impact of digitalization on healthcare infrastructural sustainability and its sharing among the stakeholders.

A cost–benefit analysis, inspired by a generalized real case and consistent with the methodology of the study, will test some of the hypotheses (Section 6.1.). It will be shown that digitalization produces tangible cost savings, so improving economic and financial margins. Sensitivity analysis applied to an empirical case will support the theoretical framework, consistent with the research question.

### 6.1. The Cost–Benefit Analysis of Digital Health

According to Rahimi [104], technological progress is widely seen as the most important driver of the rise in healthcare spending [3]. One obvious reason is that technological advances tend to be costly. For instance, magnetic resonance imaging will inevitably be more expensive than its alternative, which is usually either no test at all or a cheaper, but less accurate, diagnostic technique. Even if such technologies are shown to be cost-effective, on average, they would still be expected to increase healthcare spending because the methods of analyzing cost-effectiveness set a monetary value for health and life based on willingness to pay for it.

Perhaps digital health is different. Digital technologies often include innovative software solutions and algorithms that could be substantially cheaper than devices or drugs. These technologies also tend to focus on solutions to the notoriously inefficient delivery systems of health care globally, as opposed to the development of new treatments. Given that the alternative to digital technologies would potentially be a more labor-intensive model of care, one might expect their adoption to replace costly healthcare professional time or hospital services.

The business model is based on an interaction between pro-forma balance sheets, forecast income statements, and a combination of both to get expected cash flow statements. These accounting documents concern the SPV and include two consecutive phases: the project and construction period (usually lasting some 3 years for new hospitals) and the managerial/operational phase, typically lasting some 15–25 years (the longer, the higher the expected private revenues and, conversely, the more significant the public costs). At the end of the public concession, there is a free-of-charge transfer of the hospital to the public procurer, typically following a project–build–operate–transfer (PBOT) legal and operational scheme. Alternatives are represented by the “alphabet soup”:

- BLT: build–lease–transfer
- BOO: build–own–operate
- BOOS: build–own–operate–sell
- BOOT: build–own–operate–transfer
- BOT: build–own–transfer
- BTO: build–transfer–operate
- BRT: build–rent–transfer

The PBOT model is based on a real sample, readapted, and made anonymous. The empirical evidence that inspires the model (base case) is sourced from four PPP/PF hospital investments in Veneto Region, Northern Italy:

1. Thiene/Schio—New Hospital Complex of Santorso Santorso Hospital. Available on line: https://www.hospitalby.com/italy-hospital/santorso-hospital/ (accessed on 13 March 2020).
2. Este/Monselice—New Hospital Center for Acutes New acute-care hospital complex of monselice-este. Available online: https://www.net-italia.com/en/selezione-progetti/monselice-este-hospital/ (accessed on 13 March 2020).
3. Verona—New hospital pavilions of Borgo Trento and Borgo Roma New Verona hospital pavilions of Borgo Trento and Borgo Roma. Available online: https://www.ospedaleuniverona.it/ecm/home (accessed on 13 March 2020).
4. Treviso Ca’ Foncello—New Citadel of Health Treviso hospital. Available online: https://www.aulss2.veneto.it/ospedale/ospedale-treviso (accessed on 13 March 2020) [105].

The pilot case is built around a 3-year project and construction phase, followed by up to 25 years of public-to-private concession. Data have been standardized to prepare a realistic “template” example. All the investments are now in the managing phase and were started in the last 15 years; for this very reason, they do not explicitly incorporate digital investments.

In this model, a sensitivity analysis will consider the impact in terms of cost savings on the SPV’s income statement (within a healthcare PPP/PF). Savings range from 7.4% to 11.4% (as these are the savings indicated in the literature, as reported in the introduction) in the base case, with a worst-case scenario (cost savings from 0% to 5%) and a best-case scenario (cost savings from 12% to 20%).

It is assumed that digitalization does not have any impact on revenues. This is a very prudent hypothesis that does not consider any value co-creation profit (e.g., increased volume of treatments). Even the positive non-monetary spillover effects (improved quality of life, etc.) are not considered in the example. This leaves room for further—more comprehensive—research.

The comparison of the economic and financial margins, starting from a digital-free base case, and considering the abovementioned savings in operating expenses (opex) is synthesized in Table 2.

Opex are subtracted from the operating revenues to provide the earnings before interest and taxes (EBIT), which corresponds to the operating profit. The difference between operating revenues and monetary opex (i.e., opex excluding non-monetary depreciation and amortization) is earnings before interests, taxes, depreciation, and amortization (EBITDA). EBITDA is a key parameter since it simultaneously represents an economic and financial margin.

Opex represents the expenditure items of the cost–benefit analysis. The detail (with figures related to the base case and extendable to the further digital saving occurrences) is shown in Table 3.

Opex costs are monetary, and their impact (necessary for the introduction and absorption of digital technologies) has been included in the model.

These cumulated opex incorporate digital startup costs (undertaken by the private SPV, which can, however, benefit from economies of scale and experience, pooling resources from similar initiatives), and some initial friction (psychological, cognitive, and organizational barriers to entry), mainly from the public structure and the patients. Training and some immediate benefit for users (in the form of economic saving and/or improved performance) may represent a powerful incentive to soften unavoidable startup criticalities.

EBITDA sums up to a change in the operating net working capital (stock + receivables −payables), and a change in the capital expenditure (CAPEX, representing net tangible and intangible assets, also incorporating digital investments) provides the operating (unlevered or debt-free) cash flow. The levered cash flow corresponds to the net (free) cash flow to equity, calculated from the unlevered cash flow after deducting debt service.

All the values indicated above, starting from the base case (and so even in the absence of digitalization benefits), are positive. The results show that the investment is always economically and financially sustainable.

**Figure 2:**
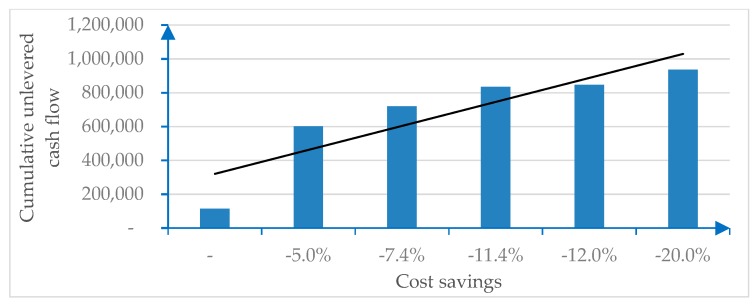
Cost savings due to digitalization.

Figure 2 is sourced from the numerical example and illustrates the sensitivity of the cost savings generated by digitalization.

The economic (EBIT, EBITDA, pre-tax, and net result) and financial (unlevered or levered cash flows) incremental margins due to digitalization show significant savings, as indicated in Table 1.

The thesis of this study, consistent with the research question, is that the incremental profits or cash flows deriving from the empirical evidence should be shared by the three main node-stakeholders (as functionally represented in Figure 1):

1. The private SPV, together with its shareholders, with indirect benefits that also concern the sponsoring banks (higher margins, associated with lower volatility due to the better “mark to market” (real vs. expected outcome) performance; reduced risk and its associated cost to capital metrics; improved bankability and long-term sustainability);
2. The public actor, which can contractually share these benefits with the SPV (for instance, decreasing the cost of services and/or the availability payment, in compliance with Eurostat best practices [15], and then use part of its savings to back unprofitable investments (e.g., in “hard” technological advances that are intelligently connected with digital networks);
3. The patients, in the form of better and more affordable services that improve value for money, a key PPP/PF public sector comparator.

The empirical example has shown that the savings induced by digitalization are meaningful and reasonably quick to grasp. These findings back the research question, indicating some useful policy recommendations for the public and private PPP stakeholders.

The ultimate benefit of the patients should never be underestimated or neglected. This is a primary target of the public intervention, within a universal healthcare coverage model that is applicable in most advanced economies.

The overall sustainability of the healthcare ecosystem can be substantially improved by digital investments, especially if they can contribute to igniting a friendly pro-growth environment. And even the synergistic interaction between digital and “hard” investments (that can be partially digitized and sponsored by digital savings) should not be underestimated.

## 7. Discussion

The main findings of the empirical case, consistent with the study purpose and research question, will be briefly analyzed in this section. Some policy implications (P4P sharing of digital savings; impact of digitalization on PPP contracts) will also be considered.

In Section 6, the empirical case has shown that digital savings improve the financial and economic parameters of the SPV. A preliminary consideration concerns the typical features and the “Galilean” replicability of the case analyzed. As shown in Section 4, the model case is inspired by four empirical realities of big healthcare PPP infrastructures in Northern Italy. This template can be easily extended to other international cases, since PPP initiatives like those considered here show consistent similarities. This is due also to European legislation, according to which big EU tenders are open to international competitors that require standard rules. The basic model is therefore easily generalizable to other healthcare PPP investments. What is still controversial is the effective impact of digitalization, due to the lack of a sufficient track record and to the intrinsic difficulty of forecasting the effect of technological innovation. 

A further issue is represented by the generalization to other industries that use PPP instruments and their technological upgrades. Whereas the overall methodology of the study—focused on a sensitivity analysis of the impact of digitalization on revenues and costs—can be generalized to different infrastructural investments that use PPP/PF patterns, some important adaptation seems necessary. First, the supply chain bottlenecks that increase costs and reduce efficiency, productivity, and value for money, exemplified in Table 1 for the peculiar healthcare case, need to be tailor-made for other occurrences that are typically very different. Additionally, the economic and financial plan, exemplified in Table 2 (and with a detail of opex items in Table 3), needs careful personalization, reflecting the nature of heterogeneous business plans. Power plants, toll bridges or other traditional PPP/PF investments are very different from hospitals.

The results that derive from the empirical healthcare case and the sensitivity analysis considered in this study are meaningful and show a consistent increase in all the profitability parameters (higher net present value and internal rate of return; lower cost of capital/Weighted Average Cost of Capital (WACC) and payback period, etc.). The WACC is a discount factor of operating cash flows, sensitive to risk reductions induced by digitalization. Additionally, the results may consistently improve, also considering the potential impact of digital scalability on revenues.

The consequences of this augmented profitability, driven by scalable digitalization, ignite a domino effect that involves all the stakeholders depicted in Figure 1 (starting from the private and the public players, but also involving the patients, the SPV suppliers, and its backing banks, etc.). 

Scalability indicates the ability of a process, network, or system to handle a growing amount of work. Scalability fosters economic marginality, especially in intangible-driven businesses (like the digital extension of smart hospitals) where variable costs are negligible. Massive volumes may offset low margins, producing economic gains. 

Digitalization is defined as the concept of “going paperless”—the technical process of transforming analog information or physical products into digital form. Digital scalability operates in a web context, where networked agents interact to generate co-created value [106]. Digital health interventions have enormous potential as scalable tools to improve health and healthcare delivery by enhancing effectiveness, efficiency, accessibility, safety, and personalization [107].

These general concepts may be conveniently adapted to the peculiar healthcare industry, where volumes (e.g., the quantity of care, lab exams, etc.) are not the final target of a patient-centric approach, where quality and timeliness of care prevail. However, volumes also refer to purchasing groups driven by B2B digital platforms, where public players are consortium members, to share savings. Additionally, volumes refer to big data that fuel patient-centric feedback, fostering economies of experience. Digital scalability therefore matters even in the distinctive healthcare framework.

Another under-investigated issue is represented by digital healthcare ecosystems. Healthcare ecosystems are increasingly patient-centric, being nurtured by growing amounts of (big) data always in need of validation. Blockchains play a vital role in this process, enabling transactions among peer-to-peer (P2P) entities without the need for a trusted third party, adding value to certified data. Patients represent a primary stakeholder that is linked to the others through an interactive network [108].

Should these digital benefits accrue only to the private stakeholder (as they represent the assignees of the SPV’s incremental proceeds), there would be no public savings to share with the public actor and the patients. This unwanted side effect would contrast with the patient-centric trend that is becoming an important goal for healthcare strategies.

Some consequential considerations may soften these concerns:

1. If private benefits lead to undeserved rents, competition grows, and private gains are reduced till a (lower) equilibrium is reached; this occurs in the tender phase, before the adjudication of the public investment to the best private competitor, who should incorporate in his offer a higher value for money, represented by better quality at a lower cost.
2. The improved quality of care immediately accrues to patients and brings to better health conditions and consequent savings on future care. Digitalization (with its mHealth applications) eases the transformation of (non-acute) inpatients into outpatients or even home patients, as shown in [81], reducing expensive and painful hospitalization rates;
3. The public actor, in the absence of shared public–private benefits, may be tempted to follow alternative ways (for instance, considering traditional procurement or public leasing, where gains are internalized, and not shared with the private partner, albeit the technological expertise of the latter would be less valuable);
4. The sharing of the digital savings should be provided for in the public–private contract, with incentives that accrue to both the counterparts and to their backing stakeholders (the patients behind the public and the banks and suppliers behind the private). These incentives may impact on the availability payment or performance fees, following a P4P approach;
5. The investment pattern typically being long term (envisaging some 3 years of the project and construction, followed by 15–25 years of management of the hospital, as shown in the empirical case), timely milestones are helpful for periodic monitoring of the (digitally-improved) performance;
6. If sharing of the digitally driven savings and efficiency gains fairly concerns the main stakeholders (the private investor and her backing banks, the public procurer, and the patients), then there is an incentive to co-create value, igniting a win–win pattern;
7. Part of the saving that accrues to the public player may be set aside to finance less profitable investments (e.g., expensive diagnostic technologies; hospitals in uneasy locations; low-income patients; orphan pathologies, etc.), to the ultimate benefit of neglected patients.
8. New investments covered by digital savings also generate opportunities for private actors, promoting sustainable economic growth. New healthcare projects are often hampered by shrinking budgets [1,2,3]. Savings and value co-creation are therefore crucial for the sponsoring of new initiatives.

It seems, therefore, evident that optimal partaking policies (risk and reward sharing with P4P patterns) actively contribute to strengthening the healthcare ecosystem. Some digital therapeutics providers accordingly offer P4P contracts [64].

A further consideration may concern tax incentives. Taxpayers have already been mentioned in Section 5., and intersect with patients, as shown in links 17 to 20, reported in the legend of Figure 1. However, tax relief may represent a further public-to-private incentive to invest in technology. A double-tax-rate system may be foreseen, for instance introducing milder taxation (in the form of tax credits for R&D expenditure) on the incremental private revenues that derive from digital/technological investments. Lower public tax revenues may be easily compensated by the public–private sharing of the additional digital gains, as shown earlier.

Even if there has been a growing literature, in the last twenty years, concerning P4P healthcare applications (see Section 2.2.), little if any evidence concerns the research question of this paper. This derives from the novelty of the topic and to the circumstance that only embryonic empirical evidence is by now available. This happens because healthcare PPP investments are long-term (lasting some 20–30 years), and digital investments have only recently been incorporated in their framework, hence need time for ex-post monitoring and assessment. Only time will tell, even if the insights of this study may help conception of an innovative legal context where digital savings can be equitably shared.

This is the case particularly in the management (operational) phase, even if also the construction phase must be “smart” and incorporate digital savings since inception. Smooth matching between the construction and the management phase—even thanks to digitalization—reduces running costs and improves efficiency and resilience, backing the overall sustainability of the investment.

The statistical treatment of PPP contracts [15] indicates some best practices that may also be used in the P4P/RBF sharing of the digital rents. Table 4 shows how digitalization may impact on some of the main practices.

It has been shown that digital platforms represent a synergistic bridging node where different stakeholders interact to co-create shareable value. This is particularly important in an innovative patient-centric scenario, where infrastructural healthcare investments are increasingly digitized and networked. A second theme of this study, consistent with the research question, is the impact of digitalization that can foster networking interaction among the PPP stakeholders. This under-investigated aspect needs further research.

PPP interactions go beyond the traditional public–private confrontation, including patients and a new “virtual” stakeholder represented by the digital platform. The digital nature of the platforms enhances their networking attitudes, leveraging scalable profitability.

## 8. Conclusions

This study concentrates on the impact of digitalization [109] on healthcare infrastructural investments. Whereas diagnostic or other “physical” technologies often increase the cost of the investment, digital applications are cheaper and may allow for timely cost reduction. They may therefore fit with the needs of public authorities that face compelling budget pressures.

To the extent that digital investments are synergic with other innovative devices (e.g., big data deriving from IoT applied to innovative magnetic resonance imaging), they can improve the overall value for money of the investment—a key parameter in project finance selection. Digital platforms are consistent with health issues, and the complexity of the stakeholders’ interaction.

In a PPP agreement, digital savings should be shared by the main stakeholders that participate in the initiative: the private actor (SPV) and his backing lenders (banks) but also the public procurer and, primarily, the ultimate beneficiaries, represented by the patients. This strategic target is consistent with a patient-centric approach and with popular eHealth or mHealth applications. Any temptation to accumulate undeserved or excessive private rents should be tamed by the competition among the private bidders participating in a PPP tender and by P4P/RBF partitioning schemes, as shown in [6] and [81]. 

Disrupting trends impact the healthcare ecosystem and concern:

1. An increasingly patient-centric vision, consistent with personalized medicine;
2. A closer interaction between actors that are traditionally part of the healthcare supply and value chain (the patients; the public universal healthcare provider, whenever present; the private investors and suppliers; etc.);
3. Augmented use of digital technologies that make healthcare services cheaper, and more readily available, consistently improving Value for Money in PPP agreements;
4. The entry of disruptive and non-conventional competitors (MedTech firms; m-app developers, etc.);
5. The demand for more sophisticated care delivery services [110] and sites, trying to transform, whenever possible (e.g., whenever acute treatment is unnecessary), inpatients into outpatients and eventually home patients;
6. Big data that are continuously created by wearables, etc., and fuel eHealth or mHealth applications, fostering value co-creation and easing patient-centricity;
7. revamped payment and public funding models, increasingly following P4P/RBF patterns and trying to optimize the trade-off between Traditional Procurement (TP) and PPP;
8. a digitally networked reinterpretation of analogic stakeholder interactions.

This study has shown that many healthcare bottlenecks can be softened with digital investments that foster long-term sustainability. New technologies and telemedicine/mHealth are useful even for infectious disease surveillance [111]. They might therefore be applied in situations like that of the Covid-19 coronavirus pandemic to support massive healthcare intervention, decongesting hospitals and providing timely big data. Digitalization is also consistent with the real-time geo-localization (through smartphone tracking or other devices) of potentially contagious individuals, softening lethal viral networking. New research avenues should concentrate on these vital issues.

The empirical example, with its sensitivity analysis, has shown that important savings can be achieved thanks to digitalization, supporting the research question. This can generate extra profits that produce incremental economic and financial margins. Additional marginality can then be shared between the main stakeholders that participate in the PPP initiative (the private SPV and its backing banks; the public player and the patients as ultimate beneficiaries). Higher margins reduce risk (represented by the WACC) and ease the bankability of traditionally capital-intensive PF healthcare investments. Sharing of these benefits may conveniently occur via P4P/RBF, incentivizing mechanisms that ignite value co-creation patterns. Whereas P4P applications have long been applied to healthcare issues, the peculiarities of this case, considering the scalable impact of digitalization [96], are still subject to pioneering investigation.

The analogic extension of this template to other cases is possible, as illustrated in the discussion. Whereas the interpretation of smart healthcare PPP investments [6,16,19] should adequately incorporate the (hardly predictable) impact of digital innovation, other industries require a preliminary definition of their business model and supply chain bottlenecks, considering the base case and the digital upgrade. This does not seem an easy task. 

This sophisticated framework requires multilevel analysis interpretation, and its evolving boundaries are too wide to be holistically considered in this study. There is room for further interdisciplinary research on this popular topic, remembering that complex issues require lively interpretation and unconventional scrutiny.

## Figures and Tables

**Table 1:** Value co-creation and softening of supply chain bottlenecks through a digital healthcare platform.

Supply Chain Bottleneck	Description	Proposed Solution/Mitigation Strategy
Last-mile unavailability: difficulties in delivering health services	Challenges in infrastructure (e.g., inadequate roads, etc.), people (e.g., lack of necessary competencies and accountability), and processes create last-mile barriers and limit access to essential health services.	Forecast analysis—digital platform communication hotspots/main health centers to bypass infrastructural drawbacks. Technologies and tools that enable effective and efficient delivery to the last mile. Long-term infrastructure planning based on data analysis (Spatial Decision Support System).
First-mile (health center) data shortage	Multiple barriers limit the efficient collection and reporting of critical health supply chain data in the first mile. These include limitations in scalable tools and platforms that efficiently capture and transmit data; overburdened staff; and poor-quality data control.	Switch to digital data acquisition; mobile apps for data acquisition at the point of care. Introduction of a standard for data recording, storing, and sharing. Innovative solutions: end-to-end supply chain visibility, data-driven forecast analysis for resource allocation.
Paper / non-digital data	Not digitized data cannot be transferred via digital platforms, and interpretation is severely impaired.	OCR software, artificial intelligence, and semantic analysis.
Data-driven performance management	Integration and analysis of data from multiple sources and triangulation of data remain challenging; data are rarely used systematically to inform decision- and policymaking.	Approaches, tools or technologies that can support data analysis and data-driven decisions and actions to improve supply chain performance.
Governance and accountability drawbacks	Formal and informal incentives in public health supply chain systems and the workforce that manages them can be misaligned to public health goals at multiple levels (from warehouse and clinic staff to policymakers). This can lead to inaction, poor decision making, or rent-seeking behaviors.	Systems or frameworks that will better align public health supply chain incentives (at the individual, organizational, or systemic level) with public health goals. Technological or system innovations reduce corruption, wastage, and leakage in the supply chain.
Sustainable human capacity-local capacity building	Massive investments in training and capacity building for supply chain management have, in many countries, failed to produce efficient operations. Public health supply chains often face difficulties in developing, attracting, and retaining qualified staff.	Innovative means for developing local supply chain technical and managerial capacity through partnerships with the private sector. Mechanisms for improving staff motivation and human resource performance management within the supply chain.
Resource mobilization and supply chain operations financing	Enough funds are not allocated for or expended on critical supply chain operations, including data distribution and collection, monitoring, and performance improvement. Data on the actual costs to operate the supply chain are rarely known within the public sector.	Innovative mobile technologies, tools, mechanisms, and approaches to ensure funds are available to overcome public challenges, such as delayed public fund transfers and low liquidity in countries.
Lack of integrated diagnostic services	Functioning of existing lab services remains poor due to low instrument utilization rates, poor data management, human resource challenges, low rates of results returned, inadequate quality systems, poor sample transportation systems, and low-quality specimens. Obstacles include connectivity; sample collection and specimen processing; sample transportation and distribution.	Optimize transportation networks, and leverage distribution capabilities from other local services to improve sample transport logistics, timelines, and cost. Adapt selective centralized laboratory instrument platforms. Seek novel ways to implement interconnected laboratory networks that will efficiently track patients, specimens, and data.

**Table 2:** Impact of digitalization on the economic and financial margins of a healthcare PPP investment (data in €/000).

Economic & Financial Plan Cases Comparison						
[data in €/000]						
	Base case					
Impact of digitalization on the operating costs	0%	−5%	−7.4%	−11.4%	−12.0%	−20.0%
Total operating revenues (3+25 years)	1.094.615					
Total operating costs (3+25 years)	885.106	395.038	277.222	161.393	149.577	60.394
Total EBIT (3+25 years)	154.243	644.314	762.130	877.962	889.778	978.964
Total pre-tax result (3+25 years)	114.628	604.766	722.613	838.494	850.317	939.593
Total net result (3+25 years)	79.954	423.336	505.829	586.946	595.222	657.715
Cumulative EBITDA (3+25 years)	209.508	699.577	817.392	933.222	945.037	1.034.221
Cumulative unlevered cash flow (3+25 years)	113.234	601.580	719.111	834.743	846.545	935.665
Cumulative levered cash flow (3+25 years)	16.125	40.331	44.332	47.118	47.321	48.248
NPV equity	17.230	115.290	140.496	167.245	170.158	194.250
NPV project	30.034	178.942	217.521	258.628	263.120	300.473
Payback Period	2029	2026	2024	2023	2023	2023
Average Debt Service Cover Ratio	2,02	6,28	7,41	8,58	8,71	9,67
IRR equity	11,66%	25,64%	28,54%	31,83%	32,22%	35,82%
IRR project	10,91%	22,69%	25,47%	28,83%	29,25%	33,35%
Average EBITDA / financial charges	11,01	41,31	47,49	52,73	53,19	56,12

**Table 3:** Opex detail (data in €/000).

Opex Detail [Data in €/000]	
	Base case 2017–2044
**Services Costs**	
Laboratory	274.789
Imaging	126.403
Housekeeping	98.924
Data Process	32.059
Security	16.487
Catering	5.496
Patient Guilding / Secretariat	25.647
Other Services	8.427
Catering Costs for Personnel and Patients	76.941
Sterilization and Disinfection	15.388
Landscaping	3.664
**Total Services Costs (A)**	**684.226**
**General SPV Annual Costs (B)**	**17.688**
Commercial Costs	
Parking Lot	20.151
Hotel and Congress Center	17.220
Shopping Mall/Center	45.798
Cafeterias and Restaurant	67.781
Nursery	9.160
Taxi Stands	23.082
**Total Commercial Costs (C)**	**183.193**
**TOTAL OPEX (D) = (A)+(B)+(C)**	**885.106**

**Table 4:** Eurostat treatment of PPP contracts and the impact of digitalization.

Theme/Contractual Provision	Impact of Digitalization
Operation and maintenance of the asset	Digitalization may improve maintenance, with real-time monitoring of its standards
Adjustments for unavailability and poor service performance	Digitalization improves availability and 24/7 monitoring, so reducing unavailability risk.
Demand-based Payments	Some PPP contracts feature demand-based payment mechanisms that calculate the Operational payments due by the authority according to the level of use of the asset. Digitalization may foster the use of non-rival intangibles.
